# Resveratrol attenuates tubular injury in diabetic kidney disease by suppressing the ferroptosis–ferritinophagy pathway

**DOI:** 10.1186/s41065-026-00718-2

**Published:** 2026-08-08

**Authors:** Dongju Zhu, Longhui Li

**Affiliations:** 1 Department of Nephrology, Affiliated Hospital of Panzhihua University, Panzhihua, Sichuan Province 617000 China; 2 Department of Nephrology, Chinese and Western Medicine Hospital of Panzhihua, Panzhihua, Sichuan Province 617000 China; 3 Department of Health Management Centre, Chongqing Academy of Medical Sciences, Chongqing General Hospital, Chongqing University, Chongqing, 401147 China

**Keywords:** Diabetic kidney disease, Resveratrol, Oxidative stress, Tubular injury, Ferroptosis, Ferritinophagy

## Abstract

**Background:**

Diabetic kidney disease (DKD) is a leading cause of end-stage renal disease, and renal oxidative stress (OS)–mediated tubular injury plays a critical role in its progression. Resveratrol (Res) possesses established antioxidative properties, yet its mechanistic involvement in ferroptosis-ferritinophagy signaling remains unclear. This study investigated the protective effects of Res on renal tubular injury in DKD and explored its regulatory role in the ferroptosis–ferritinophagy pathway.

**Methods:**

A DKD mouse model was established using unilateral nephrectomy followed by streptozotocin injection. A total of 42 male C57BL/6 N mice were used in this study. Six mice served as the blank control group, and the remaining 36 underwent model induction. The 30 successfully modeled mice were randomly divided into 5 groups (*n* = 6 per group): the DKD group, the positive control group (perindopril 0.48 mg/kg/d), and the resveratrol (Res) low-, medium-, and high-dose groups (1.0, 2.5, 5.0 mg/kg/d, administered by gavage for 8 weeks). Renal function, oxidative stress markers, and renal tubular injury markers were assessed. Hematoxylin and eosin (H&E) staining, Masson staining, 4-hydroxynonenal (4-HNE) immunofluorescence, and Western blotting (for ferroptosis/ferritinophagy-related proteins) were performed.

**Results:**

Compared with the Black group, DKD group mice exhibited marked OS, tubular injury, and activation of the ferroptosis–ferritinophagy axis. Res treatment significantly reduced fasting blood glucose, SCr, BUN, 24-hour urinary protein, MDA, Fe²⁺, β2-MG, RBP4, and NGAL levels, while elevating T-SOD and GSH-Px (*P* < 0.05). Res also downregulated Nrf2, SLC7A11, GPX4, LC3II/LC3I, and FTH1, alongside increased expression of p62 and NCOA4, demonstrating a clear dose-dependent effect.

**Conclusion:**

Resveratrol markedly attenuates renal oxidative stress and tubular injury in DKD. These findings suggest that its renoprotective effects may be mediated through inhibition of the ferroptosis–ferritinophagy pathway, highlighting Res as a promising therapeutic candidate for DKD.

**Supplementary Information:**

The online version contains supplementary material available at https://doi.org/10.1186/s41065-026-00718-2.

## Background

Diabetic kidney disease (DKD) is a frequent complication among diabetic patients and has emerged as a significant contributor to end-stage renal disease globally [1]. The pathogenesis of DKD is complex, and it is currently believed that genetics, hemodynamic changes, metabolic abnormalities, hypertension, and other factors are all key contributors to the development of DKD [2, 3]. In recent years, the incidence and mortality of DKD have increased year by year. Clinically, targeted treatments are mainly adopted based on the severity of DKD, including pharmacotherapy, end-stage renal replacement therapy, and organ transplantation, but the clinical efficacy is far from satisfactory [4]. Therefore, finding new therapeutic targets for DKD and developing new treatment measures have become hot topics in current research.

Resveratrol (Res) is a natural bioactive compound with antioxidant properties, belonging to non-flavonoid polyphenolic organic compounds. Current research has confirmed that Res has antioxidant, anti-inflammatory, anticancer, and cardiovascular protective effects [5–8]. Mahjabeen et al. found that Res supplementation helps reduce insulin resistance in patients with type 2 diabetes to improve glycemic control levels, and Res has a visible beneficial impact on chronic inflammation and oxidative stress (OS) in patients with type 2 diabetes [9]. García-Martínez et al. also showed that a daily supplement of 1,000 mg Res markedly reduces the levels of lipid peroxides and C-reactive protein in patients with type 2 diabetes and decreases the proportion of patients with severe OS [10]. Delpino et al. confirmed through a meta-analysis that Res, as a supplement in the prevention and treatment of diabetes, can markedly improve insulin resistance and reduce fasting blood glucose (FBG) and glycated hemoglobin levels [11]. In addition, Liu et al. showed through a meta-analysis that Res can improve renal function in animal models of DKD by reducing levels of serum creatinine (SCr), blood urea nitrogen (BUN), 24-hour urinary protein (24 h pro), blood glucose, and blood lipids [12]. However, the protective mechanisms of Res on tubular injury in DKD still need further exploration.

The DKD mouse model was constructed in this article to analyze the impact of different concentrations of Res on renal function, tubular injury, and renal OS response in DKD mice. This article aims to provide references for understanding the mechanisms of Res in treating DKD and for finding new therapeutic targets for DKD.

## Materials and methods

### Experimental materials

Forty-two 8-week-old male C57BL/6N mice (clean grade, healthy, body weight 20–22 g, Beijing Vital River Laboratory Animal Technology Co., Ltd., License No.: 114000700344642) were housed in the Affiliated Hospital of Panzhihua University Laboratory Animal Center under controlled conditions, which included a 12-hour light/12-hour dark cycle, ambient temperature ranging from 23 to 25 °C, and relative humidity maintained between 50% and 55%. The animals were provided with a standard laboratory diet and had unrestricted access to both food and water throughout the study period. This study strictly adhered to the recommendations of the National Institutes of Health Guide for the Care and Use of Laboratory Animals. The experimental protocol was approved by the Animal Ethics and Management Committee of the Affiliated Hospital of Panzhihua University (approval number: pending). All methods were reported in accordance with the ARRIVE guidelines 2.0 (https://arriveguidelines.org) for animal experiment reporting.

### Experimental methods

#### DKD model establishment

After one week of acclimatization, 36 mice were selected for DKD model establishment (the remaining 6 mice served as the blank control group and were not subjected to modeling). The DKD model was established using unilateral nephrectomy combined with intraperitoneal injection of streptozotocin [13]. The detailed procedures were as follows: Mice were anesthetized by intraperitoneal injection of pentobarbital sodium (50 mg/kg) (Sigma Aldrich, USA, Cat. No. P3761, purity ≥ 98%), and the right kidney was removed. Two weeks after the surgery, streptozotocin (STZ, Solarbio, China, Cat. No. S8050, purity ≥ 98% by HPLC, CAS 18883-66-4) was injected intraperitoneally at a concentration of 140 mg/kg. STZ was dissolved in citrate buffer (Solarbio, Cat. No. S8050) at pH 4.2–4.5, prepared fresh on ice. At 72 h after STZ injection, blood samples were collected from the tail tip, and fasting blood glucose was measured using an automatic biochemical analyzer (Hitachi 7020, Hitachi, Tokyo, Japan). Fasting blood glucose ≥ 16.7 mmol/L was defined as successful model establishment [14]. Among the 36 mice subjected to modeling, 30 achieved this blood glucose criterion, with blood glucose values ranging from 16.8 to 28.5 mmol/L and a mean value of 24.6 ± 3.2 mmol/L. Among the remaining mice, 4 did not meet the blood glucose criterion (modeling failure), and 2 died within 48 h after STZ injection (presumed to be due to acute STZ toxicity). Thus, the modeling success rate was 30/36 ≈ 83.3%. These 6 mice were excluded and did not proceed to subsequent grouping. 

#### Animal grouping and drug administration

The 30 successfully modeled DKD mice were randomly divided into 5 groups (*n* = 6 per group): the DKD group, the positive control group (Positive group), and the low-, medium-, and high-dose resveratrol groups (LDR, MDR, and HDR groups, respectively). In addition, a blank control group (Blank group, consisting of the 6 non-modeled mice) was included. Each group contained 6 mice as independent biological replicates. Based on a pre-experimental power analysis (24-hour urinary protein, α = 0.05, power = 0.8), a minimum of 5 mice per group was calculated to be required; considering variability and potential dropout, 6 mice were actually included in each group. The treatments for each group were as follows (all groups received an 8-week administration period, with daily gavage at 9:00 a.m. at an administration volume of 10 mL/kg):

1. Black group: Standard diet and gavage with an equal volume of normal saline.
2. DKD group: After successful modeling, mice were given gavage with an equal volume of normal saline.
3. Positive group: After successful modeling, mice were continuously given gavage with perindopril (0.48 mg/kg/d) for eight weeks. According to the conversion factor for body surface area between animals and humans (mice are approximately 12 times that of humans), the equivalent dose in mice after conversion is 0.4–1.6 mg/kg/d, and the study dose falls within this range.
4. LDR group: After successful modeling, Resveratrol (Res) (purity ≥ 98%, Sigma-Aldrich, USA, Cat. No. R5010) was administered daily by gavage.
5. MDR group: After successful modeling, Res was administered daily by gavage at a dose of 2.5 mg/kg/d.
6. HDR group: After successful modeling, Res was administered daily by gavage at a dose of 5.0 mg/kg/d. During the modeling period, insulin and other hypoglycemic drugs were not adopted.

The three doses (1.0, 2.5, and 5.0 mg/kg/d) selected in this study were based on the effective dose range (5–25 mg/kg/d) of resveratrol for ameliorating renal fibrosis in diabetic mice reported in previous studies [15]. On this basis, to further explore the protective effects and dose-response relationship at lower doses, we additionally included the 1.0 and 2.5 mg/kg/d dose groups. 

#### Renal histopathological examination

Left kidney tissues from each group of mice were collected, fixed in 4% paraformaldehyde (Cat. No. P6148, Sigma-Aldrich, USA) for 24 h, and then routinely dehydrated and embedded in paraffin to prepare 4 μm thick serial sections. Sections were subjected to H&E staining (hematoxylin and eosin, both purchased from Beijing Solarbio Science & Technology Co., Ltd., China) and Masson’s trichrome staining (kit Cat. No. HT15, Sigma-Aldrich, USA), and the procedures were performed according to the manufacturers’ instructions. Renal tubular injury (H&E) and interstitial collagen deposition (Masson) were observed under a light microscope. For each mouse, five non-overlapping fields at the corticomedullary junction (H&E, ×400 magnification) were randomly selected, and a semi-quantitative scoring system (0 = no injury, 1 = < 25%, 2 = 25%–50%, 3 = 51%–75%, 4 = > 75%) was used to assess the degree of renal tubular injury [16]; the average score was taken as the renal tubular injury score. For Masson-stained sections, five non-overlapping fields of renal interstitium (avoiding glomeruli and blood vessels) were randomly selected under ×200 magnification, and the proportion of blue positive area to the total field area was calculated using ImageJ software (version 1.53t, National Institutes of Health, USA) as the renal interstitial fibrosis index. 

#### Biochemical index detection

After drug administration, fasting blood samples were meticulously collected from the tail vein of each group to ensure accurate and consistent measurements. At 3,000 rpm for 10 min, the upper liquid was taken and used to measure FBG in the serum with an automatic biochemical analyzer (Hitachi 7020, Hitachi, Tokyo, Japan). 24 h urine was collected to measure the content of 24 h pro with a urine analyzer (Sysmex UC-3500, Sysmex Corporation, Japan). After the final drug administration, fasting blood was obtained, and the content of SCr and Blood Urea Nitrogen (BUN) was detected. 

#### Enzyme-linked immunosorbent assay (ELISA)

After collecting serum, the mice were euthanized by cervical dislocation. Kidney tissues were isolated, washed with saline (0.9% sodium chloride injection, Cat. No. S8776, Sigma-Aldrich, St. Louis, USA), and stored at -80 °C after drying. Kidney tissue homogenates were prepared and tested for OS markers (Malondialdehyde (MDA, Item Number ab238537), Total Superoxide Dismutase (T-SOD, Item Number ab65354). Glutathione Peroxidase (GSH-Px, Item Number ab102530), Fe²⁺ (Item Number ab83366)) and tubular injury markers (β2-Microglobulin (β2-MG, Item Number ab157714), Retinol Binding Protein 4 (RBP4, Item Number ab219639), Neutrophil Gelatinase-Associated Lipocalin (NGAL, Item Number ab222938)) using ELISA kits (all purchased from Abcam, Cambridge, UK). All operations were carried out strictly in accordance with the kit instructions. Absorbance was measured at a wavelength of 450 nm using a microplate reader (Multiskan FC, Thermo Fisher Scientific, Waltham, MA, USA). 

#### 4-Hydroxynonenal (4-HNE) immunofluorescence staining

Kidney tissues from each group of mice were fixed in 4% paraformaldehyde, embedded in paraffin, and sectioned into 4 μm-thick sections. The sections were dewaxed, rehydrated, and subjected to antigen retrieval, followed by blocking with 5% bovine serum albumin for 30 min. Subsequently, the sections were incubated with rabbit anti-4-HNE primary antibody (Cat. No. ab48506, Abcam, Cambridge, UK, dilution 1:200) overnight at 4 °C. They were then incubated with Alexa Fluor 488-conjugated goat anti-rabbit secondary antibody (Cat. No. ab150077, Abcam, Cambridge, UK, dilution 1:500) for 1 h at room temperature in the dark. Images were acquired under a fluorescence microscope (Olympus BX53, Olympus, Tokyo, Japan) at ×200 magnification, with five randomly selected fields per mouse. Semi-quantitative analysis was performed using ImageJ software (version 1.53t, National Institutes of Health, Bethesda, MD, USA). Results are expressed as relative fluorescence intensity and percentage of positive area (normalized to the blank control group). 

#### Western blot experiment

Kidney tissues stored at -80 °C were homogenized and lysed with Radio Immunoprecipitation Assay buffer (containing protease inhibitor, Cat. No. 89900, Thermo Fisher Scientific, Waltham, MA, USA). Protein concentration was measured with a bicinchoninic acid (BCA) kit (Item Number 23225, Thermo Fisher Scientific, Waltham, MA, USA). In this experiment, 50 µg of protein was loaded for sodium dodecyl sulfate polyacrylamide gel electrophoresis(Electrophoretic apparatus: Mini-PROTEAN Tetra, Bio-Rad, Hercules, CA, USA), and the transfer of separated proteins to a polyvinylidene fluoride membrane (Item Number 88585, Thermo Fisher Scientific, Waltham, MA, USA)was carried out, blocking the membrane with skim milk (Item Number M7409, Sigma-Aldrich, St. Louis, MO, USA)for 1 h at 25℃. Then, the sections were incubated overnight at 4 °C with primary antibodies (all purchased from Abcam, Cambridge, UK, dilution 1:1000), including anti-Nrf2 (Cat. No. ab62352), anti-SLC7A11 (Cat. No. ab175186), anti-GPX4 (Cat. No. ab125066), anti-LC3 (Cat. No. ab192890), anti-p62 (Cat. No. ab109012), anti-FTH1 (Cat. No. ab183781), anti-NCOA4 (Cat. No. ab86751), and anti-β-actin (Cat. No. ab8226). After washing, the sections were incubated with horseradish peroxidase-conjugated secondary antibody (goat anti-rabbit IgG, Cat. No. ab205718, Abcam, Cambridge, UK, dilution 1:5000) for 1 h at 25 °C. After washing, the membrane was developed with enhanced chemiluminescence (ECL) reagent (Item Number 34580, Thermo Fisher Scientific, Waltham, MA, USA) and imaged. Relative protein expression was quantified using ImageJ software (version 1.53t, National Institutes of Health, Bethesda, MD, USA), with β-actin as the reference.

### Statistical methods

All data were represented as mean ± SD, and SPSS 23.0 software (IBM Corp., Armonk, NY, USA) was employed. One-way analysis of variance (ANOVA) and the least significant difference (LSD)-t test were adopted for contrast. Post hoc pairwise comparisons were performed using Tukey’s HSD method to control the family-wise error rate. *P <* 0.05 indicated statistically meaningful differences.

## Results

### Impact of Res on FBG and 24 h pro in DKD mice

**Fig. 1:**
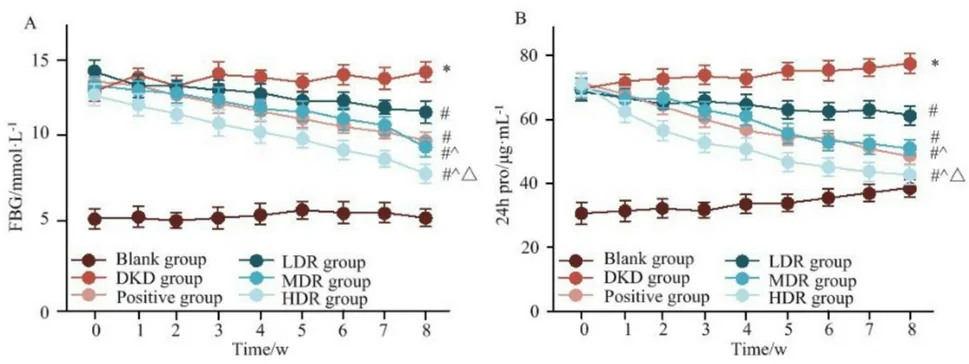
Comparison of FBG (**A**) and 24 h pro (**B**) levels. Note: One-way analysis of variance (ANOVA) followed by Tukey's HSD post hoc test was used. *As against the Black group, #as against the DKD group, ^as against the LDR group, △as against the MDR group, *P* < 0.05

In Fig. 1A, FBG levels in the Black group remained unchanged, while FBG levels in the DKD group were consistently higher as against the Black group; FBG levels in the Positive group, LDR group, MDR group, and HDR group gradually decreased and were lower as against the DKD group; FBG levels in the LDR group, MDR group, and HDR group decreased with increasing drug dosage; In Fig. 1B, 24 h pro levels in the DKD group were consistently higher as against the Black group; 24 h pro levels in the Positive group, LDR group, MDR group, and HDR group gradually decreased and were lower as against the DKD group; 24 h pro levels in the LDR group, MDR group, and HDR group decreased with increasing drug dosage (*P <* 0.05).

### Effect of resveratrol on renal histopathological changes in DKD mice

**Fig. 2:**
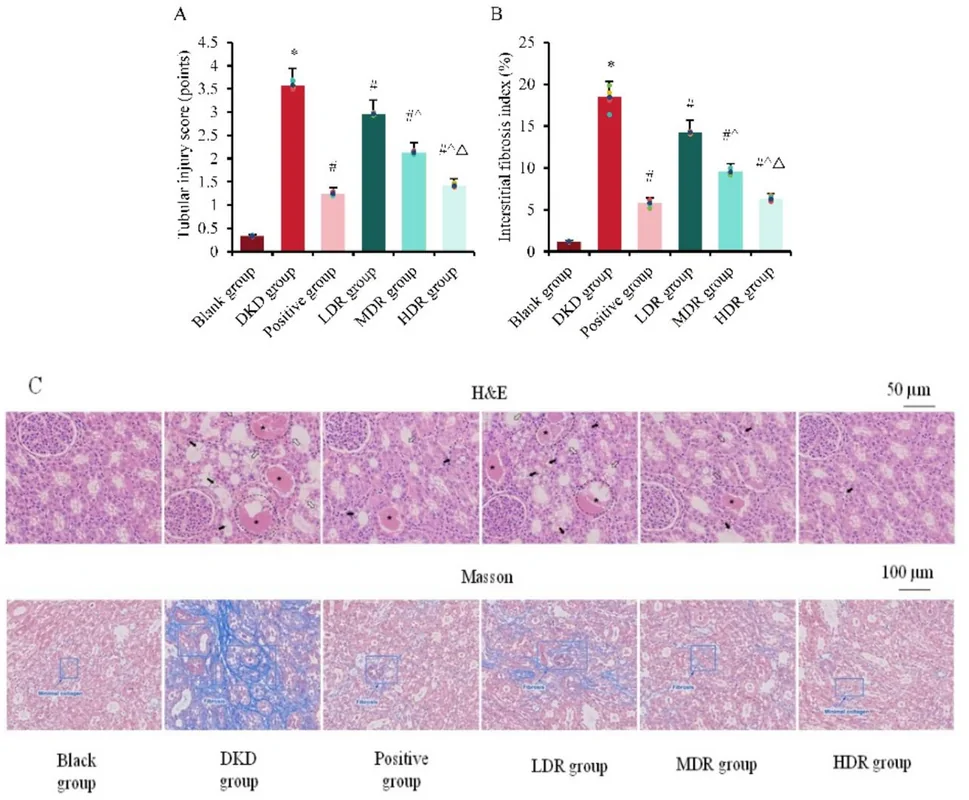
Effect of resveratrol on renal histopathology in DKD mice. Note: **A** Semi quantitative analysis of the renal tubular injury score. **B** Semi quantitative analysis of the renal interstitial fibrosis index. **C** Representative images of H&E staining of renal tissue (×400 magnification). Black solid arrows indicate vacuolar degeneration of renal tubular epithelial cells; open arrows indicate loss of brush border; asterisks (*) indicate protein casts; dashed circles indicate tubular dilatation. Representative images of Masson's trichrome staining of renal tissue (×200 magnification). Solid blue boxes indicate areas of collagen fiber deposition; blue arrows indicate interstitial fibrosis. Data are presented as mean ± SD, *n* = 6. One way analysis of variance (ANOVA) followed by Tukey's HSD post hoc test was used. * As against the Black group, #as against the DKD group, ^as against the LDR group, △as against the MDR group, *P* < 0.05

H&E staining results (Fig. 2A, C) showed that in the blank control group, the mouse renal tubules exhibited intact structure, clear epithelial cell brush borders, and no obvious vacuolar degeneration or shedding. In the DKD group, the renal tubules showed significant injury, characterized by vacuolar degeneration of tubular epithelial cells, loss of brush borders, tubular dilatation, and the presence of protein casts and epithelial cell shedding. The renal tubular injury score was significantly higher than that in the blank control group (*P* < 0.01). In the positive control group and the low-, medium-, and high-dose resveratrol groups (LDR, MDR, HDR), the degree of tubular injury was alleviated compared with the DKD group, with injury scores decreasing sequentially (*P* < 0.05). Notably, in the HDR group, the tubular structure was close to that of the positive control group, with only scattered vacuolar degeneration observed.

Masson’s trichrome staining results (Fig. 2B, C) showed that in the blank control group, only a small amount of blue collagen fibers was observed in the renal interstitium. In the DKD group, collagen fiber deposition in the renal interstitium was significantly increased, and the fibrosis index was elevated compared with the blank control group (*P* < 0.01). The degree of renal interstitial fibrosis in the positive control group and the LDR, MDR, and HDR groups was attenuated relative to the DKD group, and the fibrosis index decreased sequentially with increasing resveratrol dose (*P* < 0.05).

### Impact of Res on SCr and BUN levels in DKD mice

**Fig. 3:**
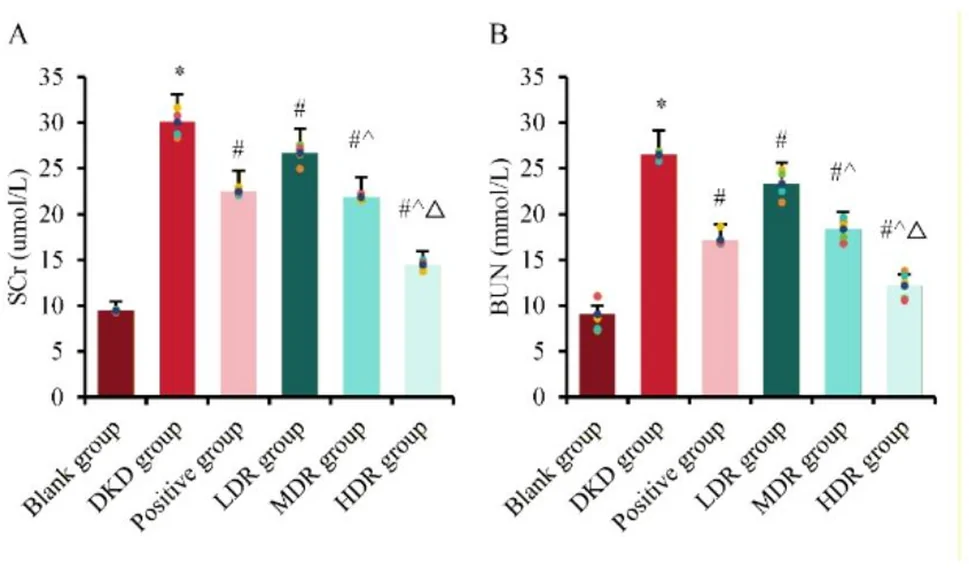
Comparison of SCr (**A**) and BUN (**B**) levels. Note: The column height represents the mean, the error bars represent the standard deviation, and each scatter point represents an independent mouse (*n* = 6 per group). One-way analysis of variance (ANOVA) followed by Tukey’s HSD post hoc test was used. *As against the Black group, #as against the DKD group, ^as against the LDR group, △as against the MDR group, *P <* 0.05

In Fig. 3A and 3B, the levels of SCr and BUN in the serum of the DKD group were higher as against the Black group; Those of the Positive group, LDR group, MDR group, and HDR group were lower as against the DKD group; Those of the LDR group, MDR group, and HDR group decreased with increasing dosage of Res (*P <* 0.05).

### Impact of Res on renal OS levels in DKD mice

**Fig. 4:**
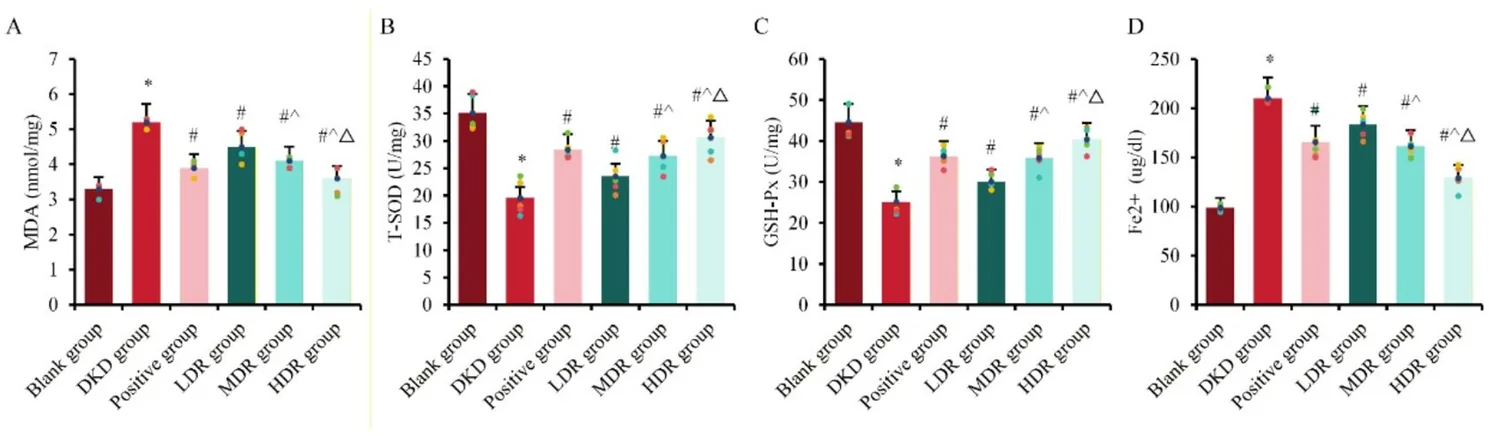
Comparison of Levels of MDA (**A**), T-SOD (**B**), GSH-Px (**C**), and Fe²⁺ (**D**). Note: The column height represents the mean, the error bars represent the standard deviation, and each scatter point represents an independent mouse (*n* = 6 per group). One-way analysis of variance (ANOVA) followed by Tukey’s HSD post hoc test was used. *As against the Black group, #as against the DKD group, ^as against the LDR group, △as against the MDR group, *P <* 0.05

In Fig. 4A and 4D, the levels of MDA and Fe²⁺ in renal tissues of the DKD group were higher as against the Black group; Those of the Positive group, LDR group, MDR group, and HDR group were lower as against the DKD group; Those of the LDR group, MDR group, and HDR group decreased with increasing dosage of Res; In Fig. 4B and 4C, the levels of T-SOD and GSH-Px in renal tissues of the DKD group were lower as against the Black group; Those of the Positive group, LDR group, MDR group, and HDR group were higher as against the DKD group; Those of the LDR group, MDR group, and HDR group increased with increasing dosage of Res (*P <* 0.05).

### Impact of Res on tubular injury in the kidneys of DKD mice

**Fig. 5:**
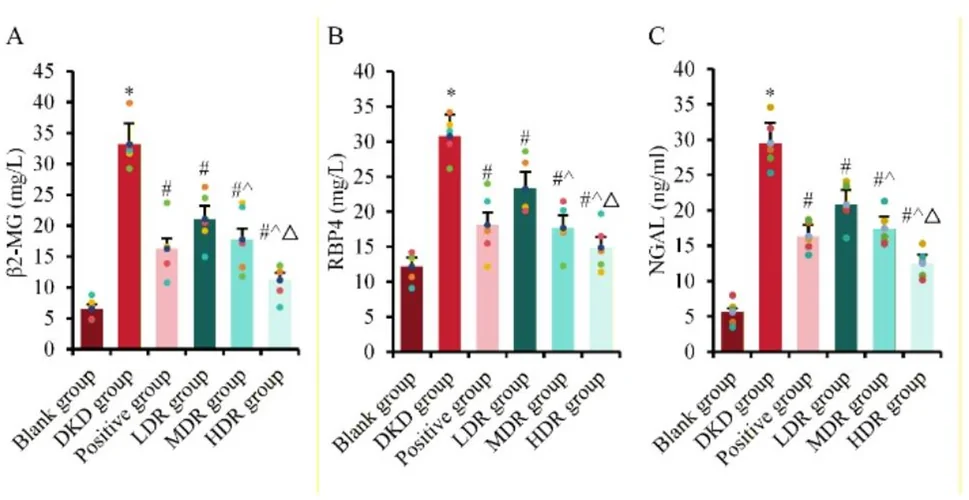
Comparison of levels of β2-MG (**A**), RBP4 (**B**), and NGAL (**C**). Note: The column height represents the mean, the error bars represent the standard deviation, and each scatter point represents an independent mouse (*n* = 6 per group). One-way analysis of variance (ANOVA) followed by Tukey’s HSD post hoc test was used. *As against the Black group, #as against the DKD group, ^as against the LDR group, △as against the MDR group, *P <* 0.05

In Fig. 5A and 5B, and 5C, the levels of β2-MG, RBP4, and NGAL in renal tissues of the DKD group were higher as against the Black group; The levels of β2-MG, RBP4, and NGAL in renal tissues of the Positive group, LDR group, MDR group, and HDR group were lower as against the DKD group; The levels of β2-MG, RBP4, and NGAL in renal tissues of the LDR group, MDR group, and HDR group decreased with increasing dosage of Res (*P <* 0.05).

### Effect of resveratrol on 4-HNE in renal tissue of DKD mice

**Fig. 6:**
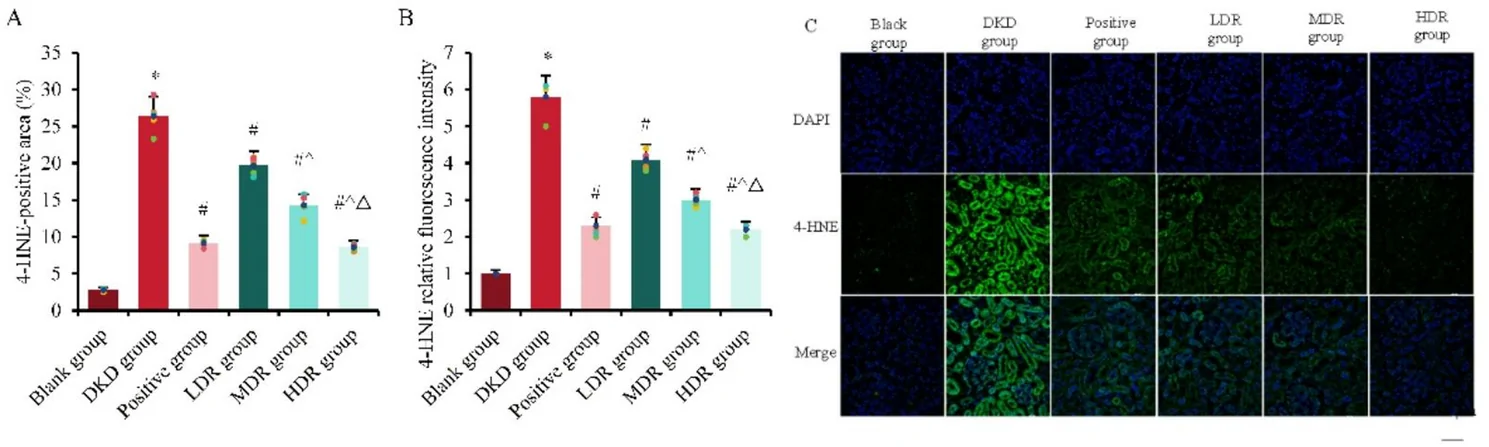
Effect of resveratrol on the expression of lipid peroxidation marker 4-HNE in renal tissue of DKD mice. Note: **A** Semi quantitative analysis of relative fluorescence intensity of 4-HNE; **B** Semi quantitative analysis of the percentage of 4-HNE positive areas; **C** Representative images of 4-HNE immunofluorescence in renal tissues of each group (green), with nuclei counterstained with DAPI (blue). The ruler is 50 µ m with a magnification of 200 times. The data is expressed as mean ± SD (*n* = 6). Adopting one-way ANOVA and Tukey HSD post hoc test. *As against the Black group, #as against the DKD group, ^as against the LDR group, △ as against the MDR group, *P* < 0.05

As shown by Fig. 6A and 6B, the 4-HNE fluorescence signal intensity and the percentage of positive area in renal tissue of the DKD group were significantly higher than those in the blank control group (*P* < 0.01). The 4-HNE fluorescence signal intensity and percentage of positive area in the positive control group and the low-, medium-, and high-dose resveratrol groups (LDR, MDR, HDR) were lower than those in the DKD group (*P* < 0.05). Moreover, in the LDR, MDR, and HDR groups, the 4-HNE fluorescence signal intensity and percentage of positive area decreased sequentially with increasing resveratrol dose (*P* < 0.05). Figure 6C shows representative 4-HNE immunofluorescence staining images of each group, which are consistent with the trend of the quantitative results.

### Impact of Res on the ferroptosis-ferritinophagy pathway in the kidneys of DKD mice

**Fig. 7:**
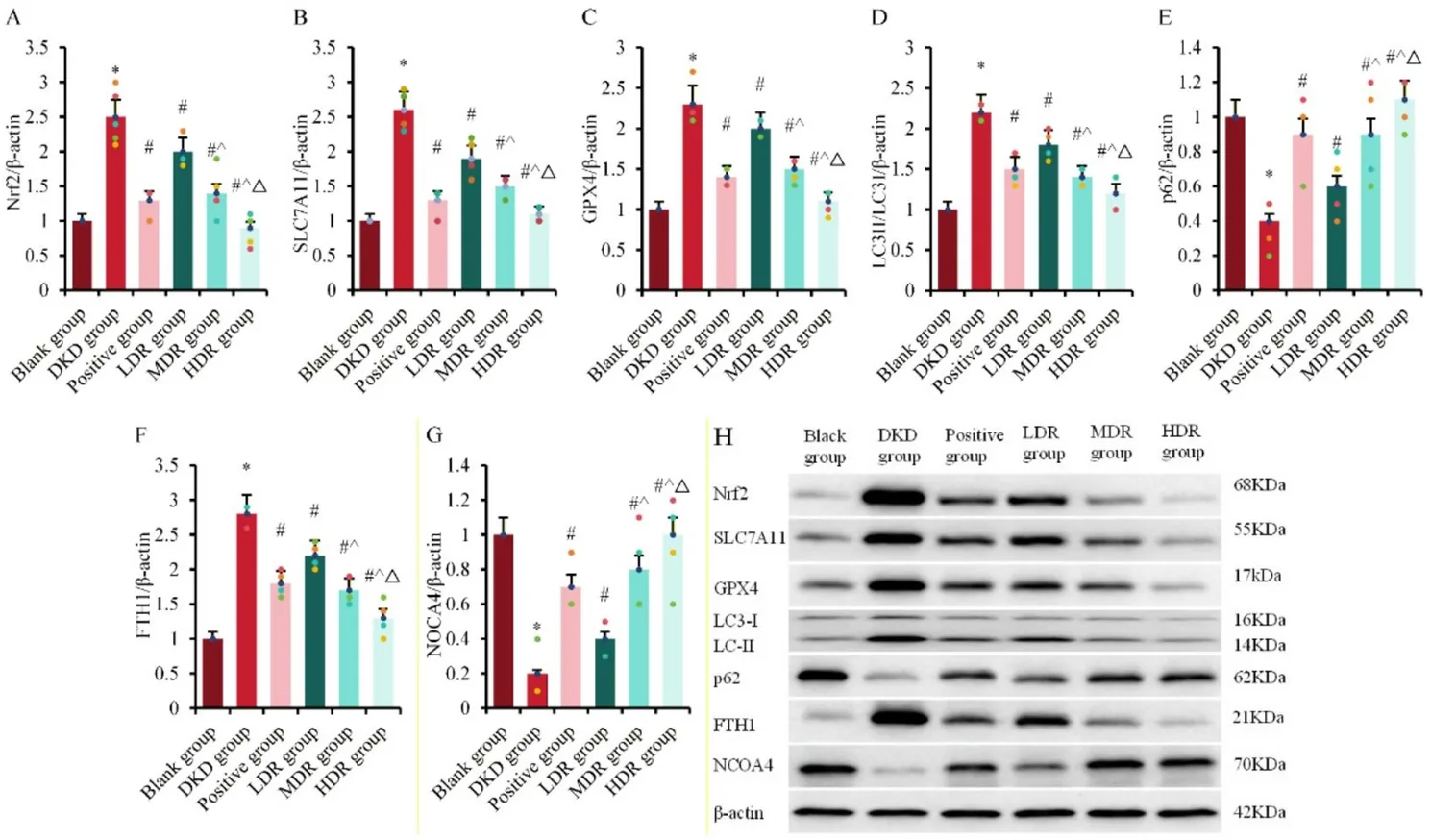
Comparison of expression levels of Nrf2 (**A**), SLC7A11 (**B**), GPX4 (**C**), LC3II/LC3I (**D**), p62 (**E**), FTH1 (**F**), and NCOA4 (**G**). Note: **H**: Representative strip images. The column height represents the mean, the error bars represent the standard deviation, and each scatter point represents an independent mouse (*n* = 6 per group). Adopting one-way ANOVA and Tukey HSD post hoc test. *As against the Black group, #as against the DKD group, ^as against the LDR group, △as against the MDR group, *P <* 0.05

In Figs. 7 A, 7B, 7C, 7D, and 7F, the relative expression (RE) of Nrf2, SLC7A11, GPX4, LC3II/LC3I, and FTH1 proteins in renal tissues of the DKD group was higher as against the Black group; The RE of these proteins in renal tissues of the Positive group, LDR group, MDR group, and HDR group was lower as against the DKD group; The RE of Nrf2, SLC7A11, GPX4, LC3II/LC3I, and FTH1 in renal tissues of the LDR group, MDR group, and HDR group decreased with increasing dosage of Res; In Fig. 7E and G, the RE of p62 and NCOA4 in renal tissues of the DKD group was lower as against the Black group; The RE of p62 and NCOA4 in renal tissues of the Positive group, LDR group, MDR group, and HDR group was higher as against the DKD group; The RE of p62 and NCOA4 in renal tissues of the LDR group, MDR group, and HDR group increased with increasing dosage of Res (*P <* 0.05). Figure 7H shows representative blot images, which are consistent with the trend of the quantitative analysis results.

## Discussion

DKD is a severe complication of diabetes, and its development is closely associated with oxidative stress. Resveratrol, a natural polyphenolic compound widely found in grapes, mulberries, and rhubarb, possesses well‑defined anti‑inflammatory and antioxidant activities [17]. Based on this, the present study investigated the protective effects of resveratrol against renal injury in DKD mice. The results showed that the three resveratrol dose groups (1.0, 2.5, and 5.0 mg/kg/day) exhibited a clear dose‑response relationship: 1.0 mg/kg/day produced moderate but significant improvements in renal function, oxidative stress, and renal tubular injury markers; 2.5 mg/kg/day further enhanced the effects; and 5.0 mg/kg/day achieved the best protective effects, with all parameters approaching those of the positive control group, and no toxicity or plateau effect was observed. This dose‑dependent protective effect may be related to the antioxidant and iron‑chelating activities of resveratrol, suggesting that higher doses could improve oxidative stress markers and modulate iron metabolism to a greater extent. These findings are highly consistent with the conclusions of a dose‑response meta‑analysis that included 42 animal studies, which confirmed that resveratrol supplementation significantly reduced blood glucose, serum creatinine, blood urea nitrogen, and 24‑hour urinary protein levels in DKD animals in a dose‑dependent manner, particularly at lower doses (≤ 15 mg/kg/day) [12]. Notably, the effective dose range (1.0–5.0 mg/kg/day) in the present study is substantially lower than the threshold reported in that meta‑analysis, suggesting that even lower doses of resveratrol exert renoprotective effects in DKD mouse models. This observation provides a preliminary reference for dose optimization in future preclinical studies. Recent studies have further revealed the multi-target effects of Res in metabolic diseases. For example, a 2023 systematic review clarified that Res can improve mitochondrial function and metabolic homeostasis by activating the SIRT1/AMPK pathway and deacetylating PGC-1α, providing a broader mechanistic explanation for its hypoglycemic and organ-protective effects in DKD [18]. These findings are consistent with the possibility that Res may lower FBG, SCr, BUN, and 24 h pro levels, which are associated with improved renal function in DKD mice.

When DKD patients present with microalbuminuria, tubular injury markers are abnormally elevated. Therefore, it is believed that tubular injury in DKD precedes glomerular damage, and some scholars have proposed the “tubulocentric hypothesis of DKD” [19, 20]. β2-MG, RBP4, and NGAL are biological markers that sensitively reflect tubular injury. β2-MG is the light chain of human leukocyte antigen class I antigens, primarily produced by lymphocytes, platelets, and polymorphonuclear proteins, and is readily filtered through the glomerular filtration membrane and reabsorbed and degraded by proximal tubule cells [21]. RBP4 is also filtered by the glomerulus and reabsorbed by the renal tubules, rarely appearing in urine. When tubular injury occurs, RBP reabsorption decreases, and urinary RBP concentration increases, making it a sensitive indicator of early tubular damage [22]. NGAL, a member of the lipocalin family, promotes the differentiation of renal progenitor cells into early tubular epithelial cells and participates in N-cadherin repair [23]. Modern studies have shown that NGAL is an effective biomarker for diagnosing acute kidney injury and can also be used for early DKD diagnosis [24, 25]. In addition to its classic role as a filtration marker, recent studies have emphasized its active role in DKD progression. For instance, evidence suggests that elevated NGAL not only reflects injury but may also participate in the fibrosis process by promoting the mesenchymal transition of renal tubular epithelial cells [26]. This article found that DKD mice exhibited increased β2-MG, RBP4, and NGAL in the kidneys, indicating visible tubular injury. This suggests that the protective effect of resveratrol may involve the renal tubular units, providing preliminary experimental support for its potential role in regulating tubule-centric pathological processes. Furthermore, Res treatment was found to decrease β2-MG, RBP4, and NGAL in the kidneys of DKD mice in a concentration-dependent manner. Histopathological examination further validated the above conclusions from a morphological perspective. In this study, H&E staining showed that resveratrol intervention dose-dependently attenuated vacuolar degeneration of renal tubular epithelial cells, loss of brush borders, and formation of protein casts in DKD mice. Masson’s trichrome staining confirmed that resveratrol significantly reduced collagen fiber deposition in the renal interstitium and decreased the interstitial fibrosis index. These morphological changes were highly consistent with the decreasing trends in renal tubular injury markers, providing intuitive histological evidence for the protective effect of resveratrol on tubular integrity. Current research indicates that OS is closely related to the occurrence and development of DKD [27, 28]. MDA, T-SOD, and GSH-Px are common indicators used to assess OS. Fe²⁺ generates hydroxyl radicals through the Fenton reaction, exacerbating OS. This article found that DKD mice exhibited increased MDA and Fe²⁺ and decreased T-SOD and GSH-Px in the kidneys. After Res treatment, MDA and Fe²⁺ decreased, while T-SOD and GSH-Px increased, similar to the findings of Hu et al. that Res markedly reduces MDA levels and restores SOD, GSH, and GSH-Px activity in DKD animal models [29].Notably, iron overload (elevated Fe²⁺) is not only a result of oxidative stress (OS) but also can reversely promote ROS generation, forming a vicious cycle. Recent views suggest that there is a profound interaction between oxidative stress and iron metabolism disorders in DKD, which together constitute a pathogenic network known as the “oxidation-ferroptosis axis” [30, 31]. The observation that resveratrol simultaneously improves oxidative stress markers and iron homeostasis is consistent with the proposed “oxidative stress-ferroptosis axis”, and suggests that this network may represent a therapeutic target.

Ferroptosis is an iron-dependent form of cell death driven by the accumulation of lipid peroxidation products in the cell membrane, characterized by impaired GPX4 function [32]. Patients with DKD often exhibit elevated levels of free Fe²⁺ in renal tubular epithelial cells, which catalyzes the Fenton reaction to produce reactive oxygen species, inducing lipid peroxidation and ferroptosis [33]. This process is regulated by key proteins such as Nrf2, SLC7A11, and GPX4. Another important source of intracellular Fe²⁺ is ferritinophagy – a selective autophagic process that releases free Fe²⁺ by degrading iron storage proteins (e.g., FTH1), thereby regulating iron metabolism [34]. LC3II/LC3I, p62, NCOA4, and FTH1 are well-recognized markers of ferritinophagy [35, 36]. When ferritinophagy is hyperactivated, excessive Fe²⁺ is released, further exacerbating the Fenton reaction and lipid peroxidation, thereby initiating or amplifying ferroptosis [37]. The present study found elevated Fe²⁺ levels in the kidneys of DKD mice, along with upregulation of Nrf2, SLC7A11, GPX4, LC3II/LC3I, and FTH1 protein expression, and downregulation of p62 and NCOA4 expression. These results suggest that changes in the expression of ferritinophagy- and ferroptosis-related proteins may be associated with aggravated renal tubular injury. It should be noted that the upregulation of GPX4, SLC7A11, and Nrf2 in DKD differs from the classical ferroptosis model, but this is not contradictory. A 2025 study in *Diabetes* confirmed that DKD mice and high-glucose/high-fat cell models also exhibit dysregulation of NCOA4 and FTH1 expression, and that inhibition of NCOA4 restored FTH1 and suppressed ferroptosis [38]; similarly, in a cisplatin-induced acute kidney injury model, decreased NCOA4 accompanied by a compensatory increase in FTH1 was observed, which was considered a cellular compensatory response to iron metabolism dysregulation [39]. Therefore, the inverse relationship between downregulated NCOA4 and upregulated FTH1, together with the compensatory activation of the Nrf2/SLC7A11/GPX4 pathway, collectively suggest a dynamic imbalance between iron metabolism and oxidative stress in DKD. Moreover, the 4-HNE assay also confirmed that lipid peroxidation was significantly enhanced in DKD renal tissue and was effectively suppressed by resveratrol.

**Fig. 8:**
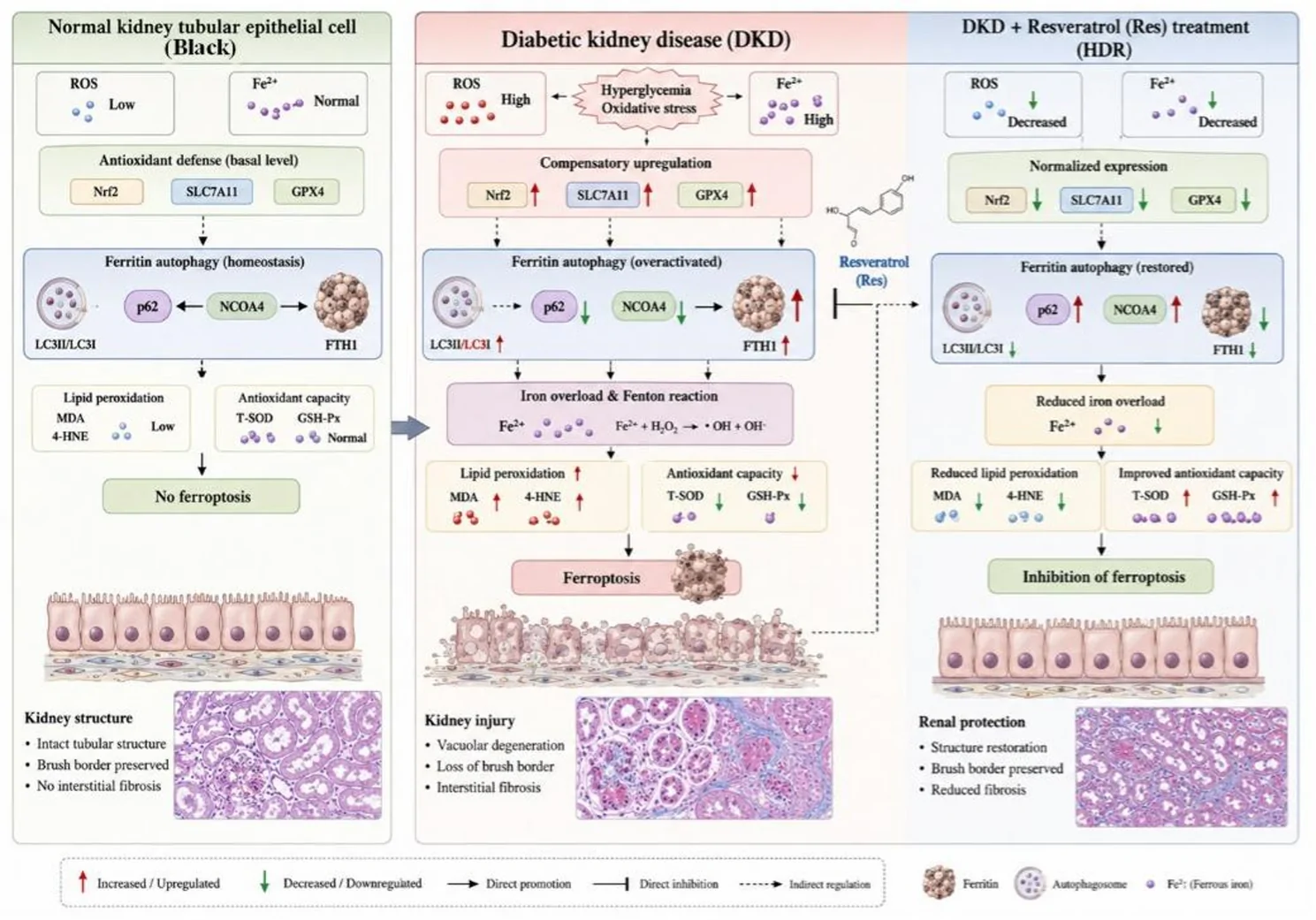
Schematic diagram of the mechanism by which resveratrol regulates the iron death ferritin autophagy pathway to protect against DKD renal tubular injury

The present study further found that after resveratrol treatment, DKD mice showed decreased Fe²⁺ levels, downregulation of Nrf2, SLC7A11, GPX4, LC3II/LC3I, and FTH1, and upregulation of p62 and NCOA4. This is consistent with the results reported by Li et al. [40] and Xu et al. [41], who showed that resveratrol inhibits the SLC7A11/GPX4 pathway or activates the Nrf2/FTH1/GPX4 pathway. Notably, after resveratrol treatment, NCOA4 was increased while FTH1 was decreased. A study on dapagliflozin showed that the DKD model baseline group exhibited an NCOA4↓/FTH1↑ pattern, suggesting abnormal activation of ferritinophagy, and drug treatment reversed this pattern [42]; the opposite effect of resveratrol in the present study further supports that the protective effect of resveratrol may be related to inhibition of excessive ferritinophagy activation. Furthermore, a 2025 study published in *World Journal of Diabetes* demonstrated that inhibition of HERC2/NCOA4-mediated autophagy-dependent ferroptosis by promoting K48-ubiquitin-mediated degradation of NCOA4 alleviated renal fibrosis in DKD [43]. These independent lines of evidence collectively indicate that regulating NCOA4-mediated ferritinophagy is an important strategy for treating DKD, and the mechanism of action of resveratrol is highly consistent with the above findings. In summary, these findings suggest that resveratrol may inhibit the activation of ferritinophagy, thereby alleviating ferroptosis in renal tissue of DKD mice, as illustrated in Fig. 8. However, the present study has certain limitations. First, although we detected enhanced lipid peroxidation by 4-HNE staining, we did not employ the gold-standard methods for detecting ferroptosis (e.g., C11-BODIPY staining or transmission electron microscopy to observe mitochondrial ultrastructure), nor did we use specific inhibitors (e.g., ferrostatin-1) for rescue experiments. Second, due to depletion of the remaining kidney tissue, we were unable to perform immunohistochemical localization of key proteins such as GPX4, Nrf2, LC3, FTH1, and NCOA4 specifically in renal tubular epithelial cells, and therefore cannot completely exclude the contribution of glomeruli or other cell types. These shortcomings may affect the robustness of the conclusions, and future studies are needed to address these issues.

## Conclusion

Res exerts protective effects against tubular injury in DKD mice, possibly by regulating the ferroptosis-ferritinophagy pathway to alleviate renal OS injury and ultimately protect renal function. The findings of this article provide insights into the mechanisms by which the ferroptosis-ferritinophagy pathway regulates the progression of DKD and offer a reference for the clinical application of Res in treating DKD.

## Supplementary Information

Supplementary Material 1.

## Data Availability

The data are available from the corresponding author on reasonable request.
