# Supplementary material for: Resveratrol attenuates tubular injury in diabetic kidney disease by suppressing the ferroptosis–ferritinophagy pathway
Source: Hereditas. 2026 Aug 8;163:126. doi: 10.1186/s41065-026-00718-2 (PMC13737594; doi:10.1186/s41065-026-00718-2)
Supplement: Supplementary file 1 [file 41065_2026_718_MOESM1_ESM.pdf]

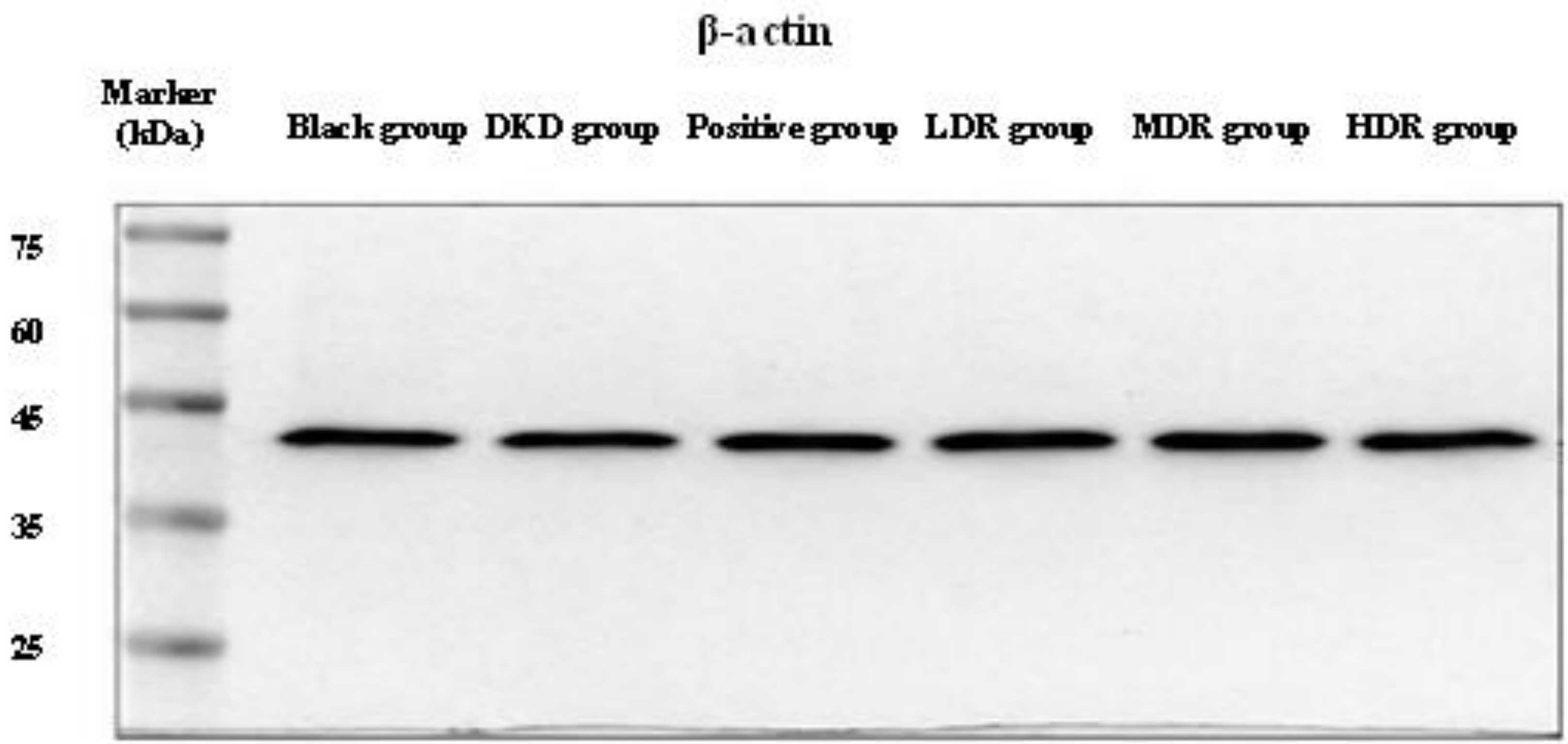
β-actin
Marker (kDa)
Black group
DKD group
Positive group
LDR group
MDR group
HDR group
75
60
45
35
25

SLC7A11

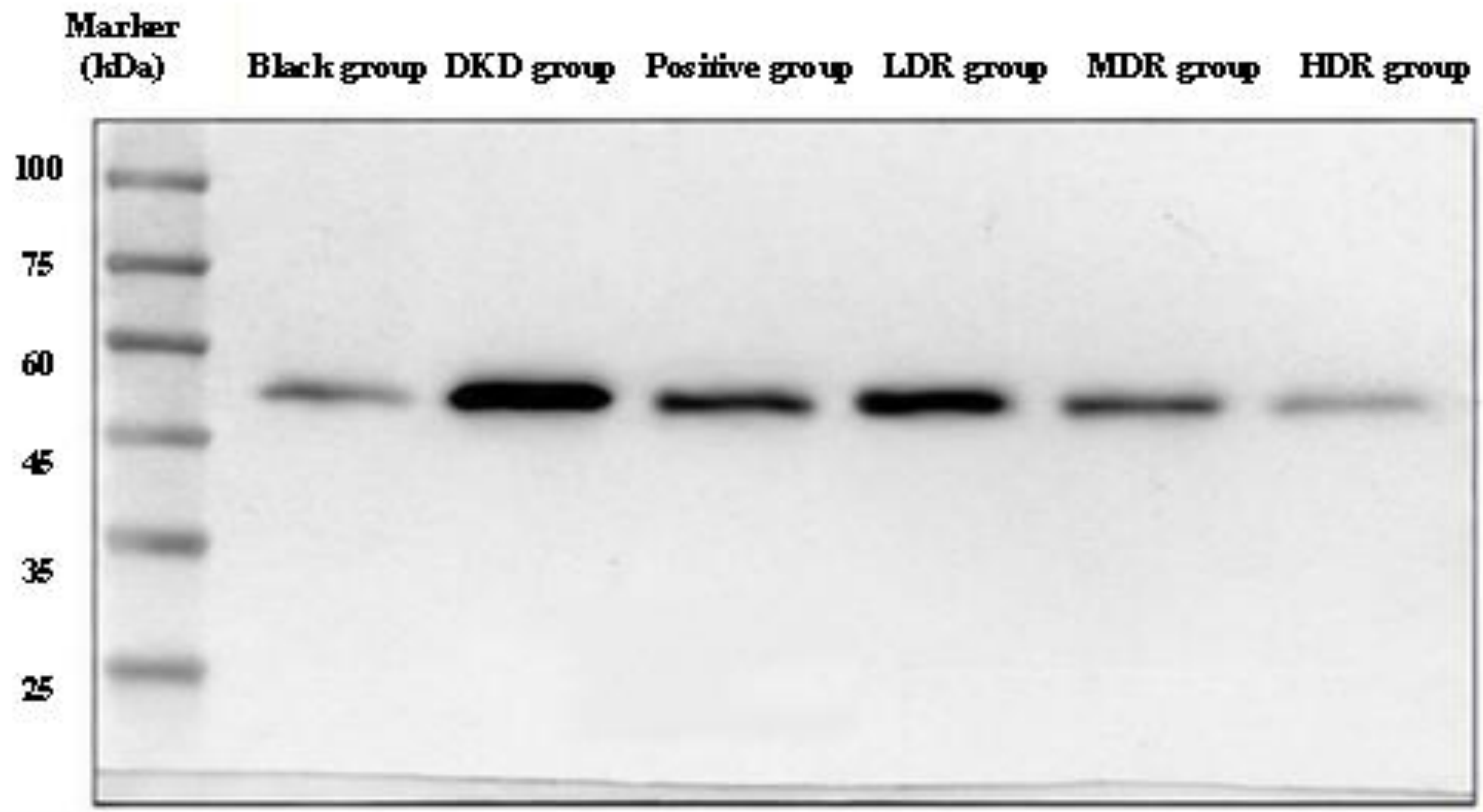
Marker (kDa)
Black group
DKD group
Positive group
LDR group
MDR group
HDR group
100
75
60
45
35
25

p62

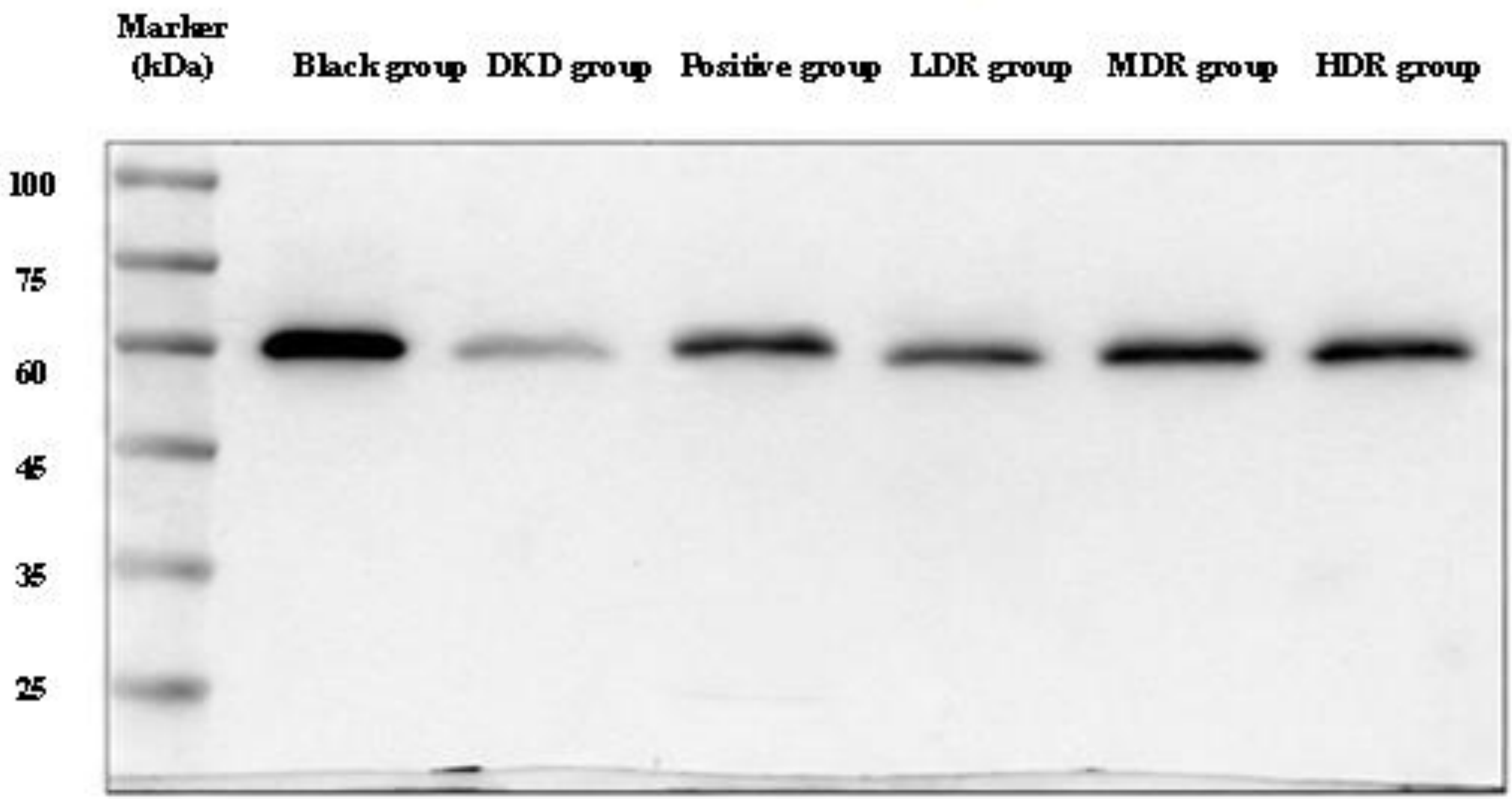
Marker (kDa)
Black group
DKD group
Positive group
LDR group
MDR group
HDR group
100
75
60
45
35
25

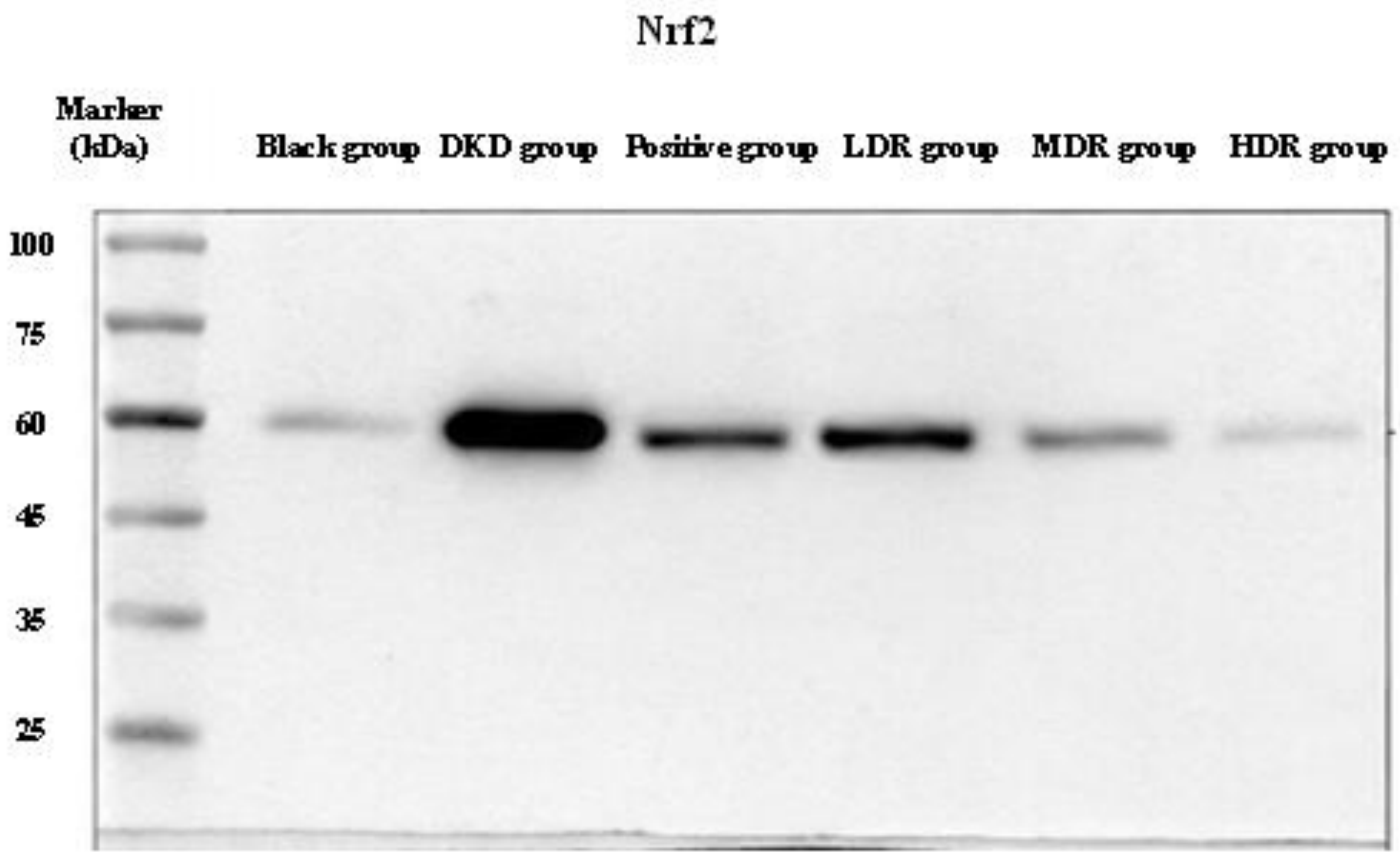
Nrf2
Marker (kDa)
Black group
DKD group
Positive group
LDR group
MDR group
HDR group
100
75
60
45
35
25

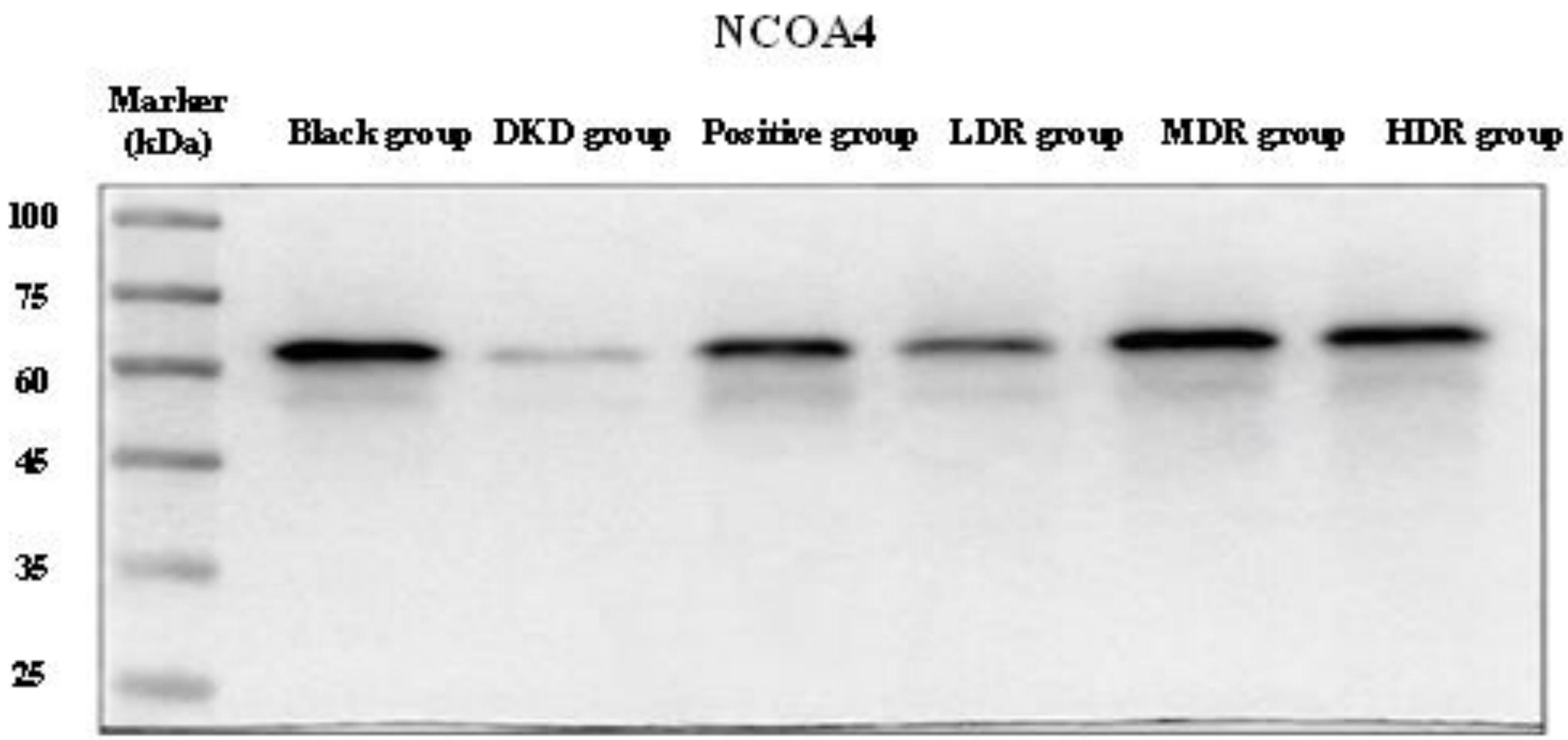
NCOA4
Marker
(kDa)
Black group
DKD group
Positive group
LDR group
MDR group
HDR group
100
75
60
45
35
25

LC3-I/II

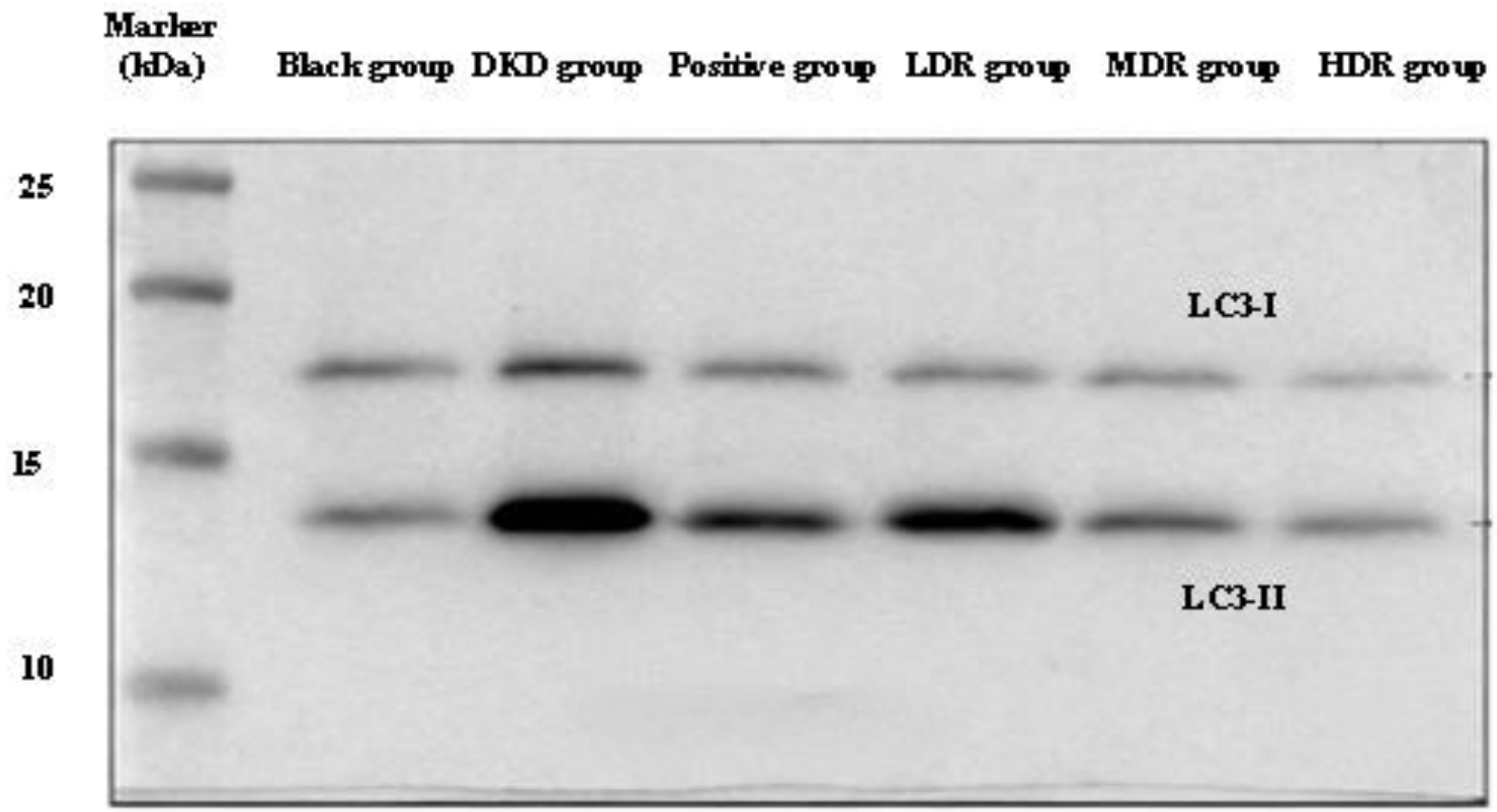

Marker (kDa)
Black group
DKD group
Positive group
LDR group
MDR group
HDR group
25
20
15
10
LC3-I
LC3-II

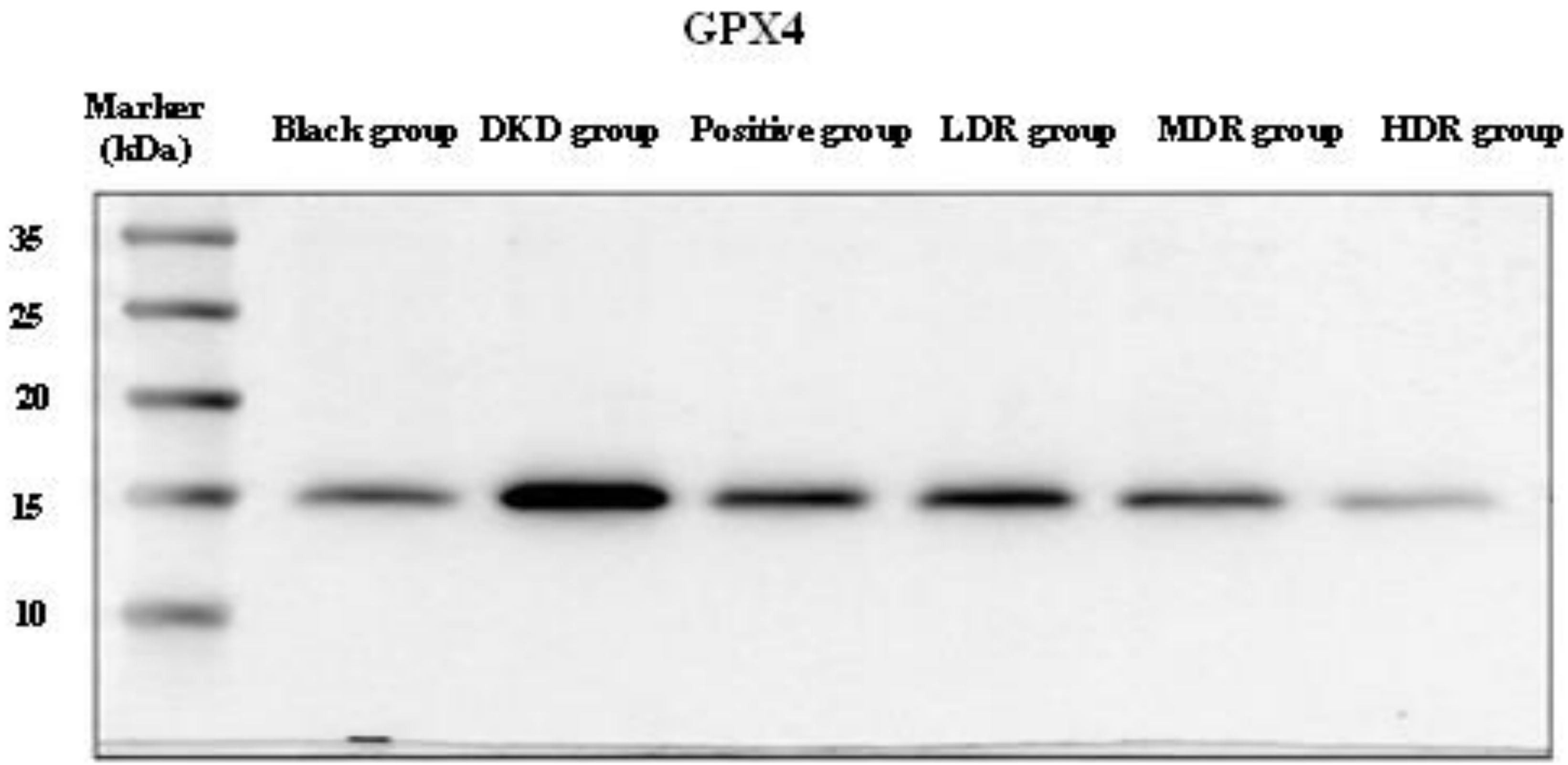
GPX4
Marker (kDa)
Black group
DKD group
Positive group
LDR group
MDR group
HDR group
35
25
20
15
10

FTH1

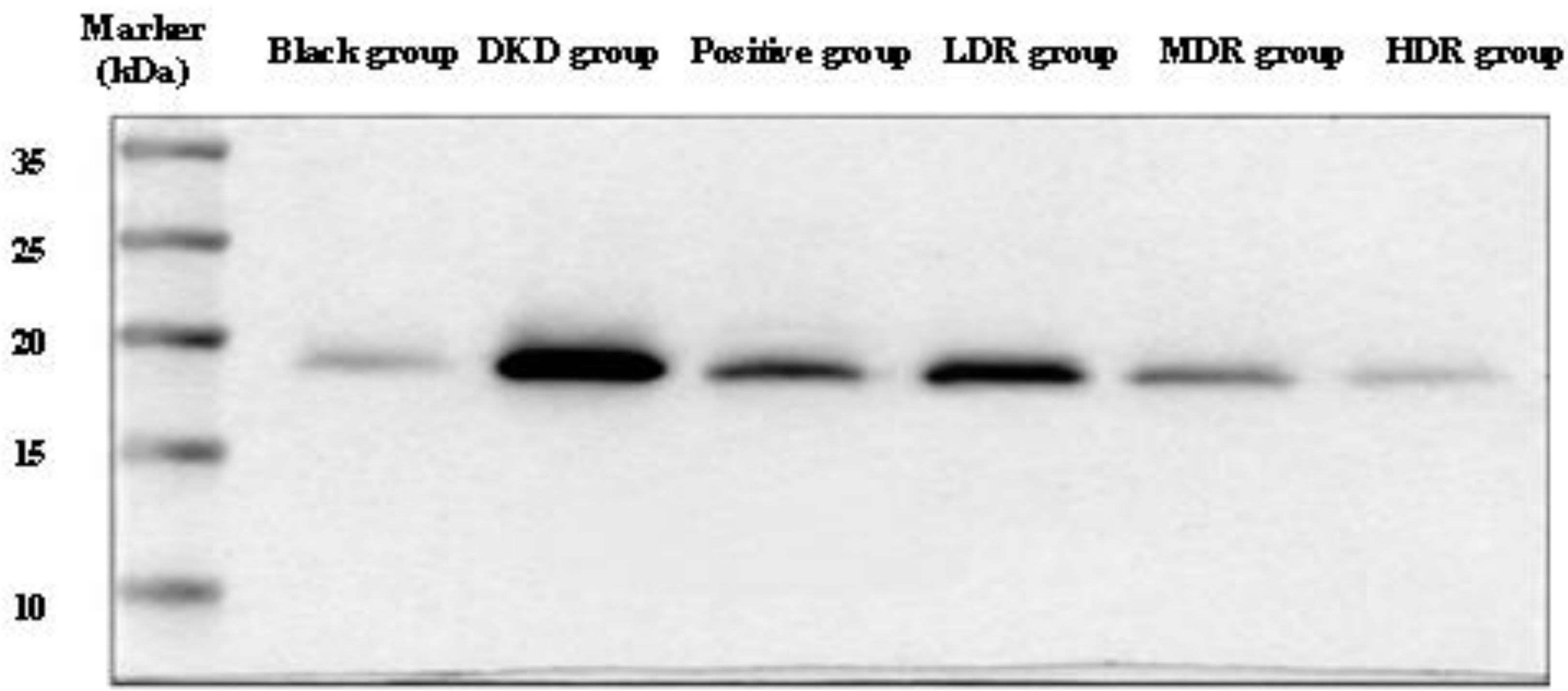
Marker (kDa)
Black group
DKD group
Positive group
LDR group
MDR group
HDR group
35
25
20
15
10
